# Immobilization of cesium from aqueous solution using nanoparticles of synthetic calcium phosphates

**DOI:** 10.1186/s13065-018-0455-9

**Published:** 2018-07-24

**Authors:** Oksana Livitska, Nataliia Strutynska, Kateryna Loza, Oleg Prymak, Yuriy Prylutskyy, Olha Livitska, Matthias Epple, Nikolai Slobodyanik

**Affiliations:** 1 Taras Shevchenko National University of Kyiv, Volodymyrska Str. 64, Kiev, 01601 Ukraine; 2 University of Duisburg-Essen, Inorganic Chemistry and Center for Nanointegration Duisburg-Essen (CeNIDE), Universitaetsstr. 5-7, 45117 Essen, Germany

**Keywords:** Cesium, Apatite, Whitlockite, Immobilization, Phosphates

## Abstract

The particularities of cesium incorporation into synthetic calcium phosphates with either apatite or whitlockite-type structures were investigated using the sorption process from aqueous solution and further heating to 700 °C. The nanoparticles for sorption were prepared by wet precipitation from aqueous solutions at a fixed molar ratio of Ca/P = 1.67 and two different ratios of CO_3_^2−^/PO_4_^3−^ (0 or 1). The obtained substituted calcium phosphates and corresponding samples after the sorption of cesium from solutions with different molar concentrations (c(Cs^+^) = 0.05, 0.1 and 0.25 mol L^−1^) were characterized by powder X-ray diffraction, FTIR spectroscopy, scanning electron microscopy and elemental analysis. Based on the combination of X-ray diffraction and elemental analyses data for the powders after sorption, the cesium incorporated in the apatite- or whitlockite-type structures and its amount increased with its concentration in the initial solution. For sodium-containing calcium phosphate even minor content of Cs^+^ in its composition significantly changed the general principle of its transformation under annealing at 700 °C with the formation of a mixture of α-Ca_3_(PO_4_)_2_ and cesium-containing apatite-related phase. The obtained results indicate the perspective of using of complex substituted calcium phosphates nanoparticles for immobilization of cesium in the stable whitlockite- or apatite-type crystal materials.

## Introduction

Calcium phosphates with apatite (Ca_10_(PO_4_)_6_(OH)_2_) and whitlockite (β-Ca_3_(PO_4_)_2_) type of structure have been extensively studied in recent years for potential applications as biomaterials [1–5], fluorescing probes [6–8], drug-carriers [9, 10], catalysts [11–13] and hosts for luminescent materials [14, 15]. Such compounds and their complex substituted analogues possess a variety of useful properties such as a high biocompatibility and bioactivity, osteoconductivity, antimicrobial effect, thermal and chemical stability [16, 17].

Among the great variety of anionic substituents, carbonate ions in the complex calcium phosphate structure affect the crystallinity of samples, their dissolution rates and the biological behavior creating lattice distortion and crystal defects. For carbonate-substituted hydroxyapatite (the general formula Ca_10–x/2_{(PO_4_)_6–x_(CO_3_)_x_}{(OH)_2–2y-_(CO_3_)_y_}], the carbonate groups can be located at two different sites depending on temperature and conditions of the sample preparation. The type A is realized when OH^−^-ions are substituted by CO_3_^2−^ ions, while in type B apatite, PO_4_^3−^-ions are substituted by CO_3_^2−^-ions. The two types of substitution, A-type and B-type, can also occur simultaneously, resulting in a mixed AB-type substitution. This more complex substitution model occurs almost exclusively in aqueous precipitation reactions [18, 19].

Besides that, the characteristic structural flexibility to accommodate variety of heterovalent ions, and resistance towards irradiation make calcium phosphate framework perspective hosts for the immobilization of radioactive waste elements [20, 21]. Such matrixes are considered to be available materials for the removal of toxic metals from polluted soils, sediments and waters, allowing rehabilitation of soils and restoration of highly polluted industrial sites. In particular, the incorporation of harmful ions such as Sr^2+^ [22], U^6+^ [23, 24], Pb^2+^ [25, 26], Th^4+^ [27], Cd^2+^ [28, 29], Ni^2+^ and Co^2+^ [30], Fe^2+^ [20] into calcium phosphate frameworks has been reported.

^137^Cs is a very hazardous nuclide, radioactive element with a high solubility of its compounds. It migrates into the environment through groundwater. Such mobility can be considerably reduced by adsorption of cesium on the rocks and minerals surrounding the nuclear waste repository. The rate of downward migration of ^137^Cs decreases with time and varies with soil type, texture, or water condition due to the fixation of element to soil particles. Most studies of cesium adsorption have been carried out on rocks, soils, sediments and minerals but only a few studies have evaluated the immobilization of cesium on the complex calcium phosphates [31–33].

The aim of the present work was to investigate the possibility of using of calcium phosphates as stable materials for immobilization of cesium. The nanoparticles of calcium phosphates with different compositions were prepared, characterized and used for sorption of Cs^+^ from aqueous solutions at different concentration.

## Experimental section

### Preparation of complex substituted calcium phosphates

On the first stage the nanoparticles of calcium phosphates which were used as initial materials for sorption of Cs^+^ were prepared by the wet precipitation method from aqueous solutions of the system M–Ca^2+^–NO_3_^−^–CO_3_^2−^–PO_4_^3−^ (M−Na, K) (at fixed molar value Ca/P = 1.67 and different ratios CO_3_^2−^/PO_4_^3−^ = 0 or 1). Ca(NO_3_)_2_·4H_2_O, M_2_CO_3_ and M_2_HPO_4_ (M−Na, K) were used as initial components. The solution with M_2_HPO_4_ or a mixture of M_2_CO_3_ and M_2_HPO_4_ (M−Na, K) was immediate added into a reactor containing Ca(NO_3_)_2_·4H_2_O. The obtained amorphous precipitates were collected by filtration and washed several times with water to eliminate any residual salts. Synthesized solids were dried at 80 °C (24 h) and then used for sorption process. All of our syntheses were performed at room temperature to prepare amorphous calcium phosphates, as increased synthesis temperatures lead to crystalline products. The powders also were heated to 400 and 700 °C for 1 h for investigation of their chemical and phase compositions.

### Sorption experiments

The 1 g of synthetic calcium phosphate under continuously stirring was added to 150 mL of aqueous solution of cesium nitrate with different concentration (0.05, 0.1 and 0.25 mol L^−1^). The heterogeneous systems were stirred for 1 h. After that the phosphates were separated by filtration, dried at 80 °C and also heated to 700 °C. All samples obtained symbols depending on their synthesis condition which are depicted in Tables 1 and 2.

**Table 1:** Indexes and chemical composition of obtained samples in system M–Ca^2+^–NO_3_^−^–CO_3_^2−^–PO_4_^3−^ (M–Na, K) (at fixed molar value of Ca/P = 1.67 and different ratio CO_3_^2−^/PO_4_^3−^) after heating to 400 °C

Samples index	M	Molar ratio CO_3_^2−^/PO_4_^3−^ in initial solution	M (wt%)	Ca (wt%)	P (wt%)	C (wt%)	(Ca + M)/(P + C) (mol)
I	Na	0	0.28	34.40	18.76	–	1.44
II		1	2.49	31.44	12.99	1.85	1.56
III	K	0	–	33.27	17.83	–	1.44
IV		1	1.72	36.87	14.14	2.34	1.48

**Table 2:** The unit cell parameters for whitlockite- and apatite-related complex substituted calcium phosphates obtained at 700 °C

Samples index	M	Molar ratio CO_3_^2−^/PO_4_^3−^ in initial solution	Crystal system	*a*, Å	*c*, Å
I	Na	0	Trigonal	10.430(8)	37.358(2)
II		1	Hexagonal	9.395(8)	6.901(5)
III	K	0	Trigonal	10.430(2)	37.391(2)
IV		1	Hexagonal	9.399(4)	6.890(1)

### Characterization of prepared samples

The phase compositions of the as-prepared powders, corresponding heated and samples after sorption were determined by X-ray diffraction (XRD). Shimadzu XRD-6000 and Bruker D8 ADVANCE diffractometers with Cu-Kα radiation were used. The data were collected over the 2*θ* range 5.0–90.0° with steps sizes of 0.02 and 0.01° and counting times of 1–2 and 0.3 s, respectively. The identification of phases was achieved by comparing the diffraction patterns of the synthesized powders with those standards of The International Centre for Diffraction Data (ICDD). The program Fullprof was used for calculation of lattice parameters.

Fourier transform infrared spectra (FTIR) were obtained using PerkinElmer Spectrum BX spectrometer in the range 400–4000 cm^−1^ (at 1 cm^−1^ resolution) for the samples pressed into the pellets of KBr.

The morphologies and shapes of the particles were observed by Scanning electron microscopy (SEM). It was performed with a FEI Quanta 400 ESEM instrument in a high vacuum after sputtering with Au:Pd or Pt. The surface composition of obtained samples was investigated using Energy-Dispersive X-ray (EDX) spectroscopy which was carried out with a Genesis 4000 instrument.

The elemental compositions of samples were determined by an atomic absorption spectroscopy instrument (Thermo Electron M-Series), X-ray fluorescence method using « Elvax Light » spectrometer and CHN elemental analysis (Elementar-Analysensysteme).

## Results and discussion

### Characterization of prepared calcium phosphates before sorption

**Fig. 1:**
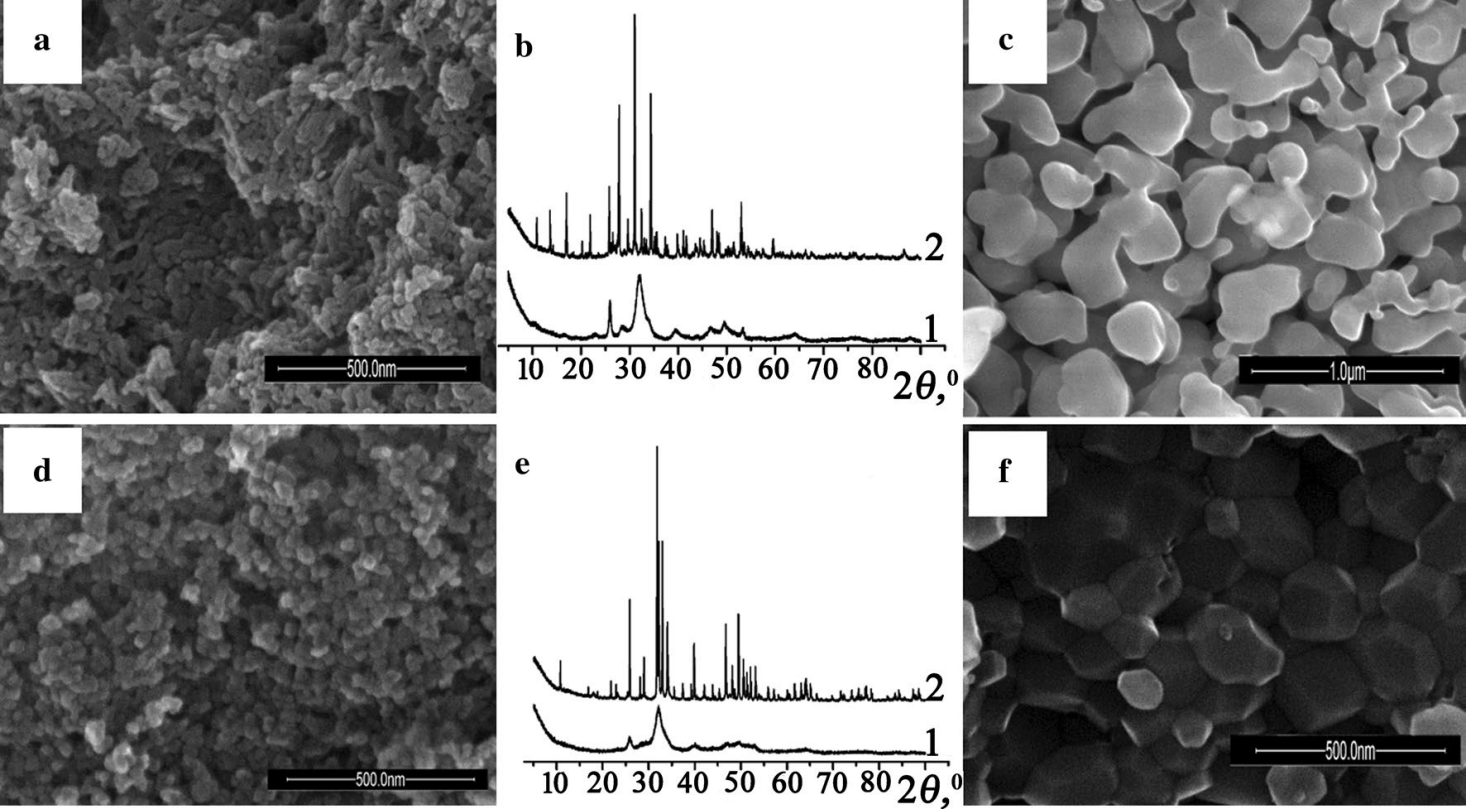
Example of SEM images (**a**, **c**, **d**, **f**) and XRD patterns (**b** and **e**) for calcium phosphates (samples III (**a**–**c**) and IV (**d**–**f**)) dried at 80 °C (**a**, **d**, curves 1) and heated to 700 °C (**c**, **f**, curves 2)

The XRD patterns of all precipitated and dried at 80 °C calcium phosphates are similar and contain broad reflections in 20–60° *2θ* ranges, which are characteristic for poorly crystalline phases (Fig. 1b, e, curves 1). According SEM data, all crystallites took shape as spherical particles and are characterized by size about 5–25 nm independently of both—type of initial phosphate component (sodium or potassium) and molar ratio CO_3_^2−^/PO_4_^3−^ = 0 or 1 (Fig. 1a, d). The values of specific surface area for initial matrix (the samples I and II) were obtained by nitrogen adsorption. The BET surface area attained the values 85 and 100 m^2^ g^−1^ for sample I and II, respectively, that corresponds to the average size of particles about 20 nm. These results correlated with SEM data. It should be noted that influence of the nature of alkaline metals on specific surface area as well as sizes and form of particles was not found.

TG/DTA data for all prepared samples are similar as early reported in [18, 19]. The TG results demonstrated the three temperature ranges of mass losing: 80–350, 450–650 and above 700 °C. The first one is attributed to elimination of adsorbed water, the second region is dealt with simultaneously CO_2_ and water losing. The last one is accompanied by the partial samples destruction [18, 19]. Based on TG/DTA results, all samples were heated to 400 °C for elimination of sorbed water and carrying out elemental analysis and then were annealed at 700 °C for determination of their phase composition.

**Fig. 2:**
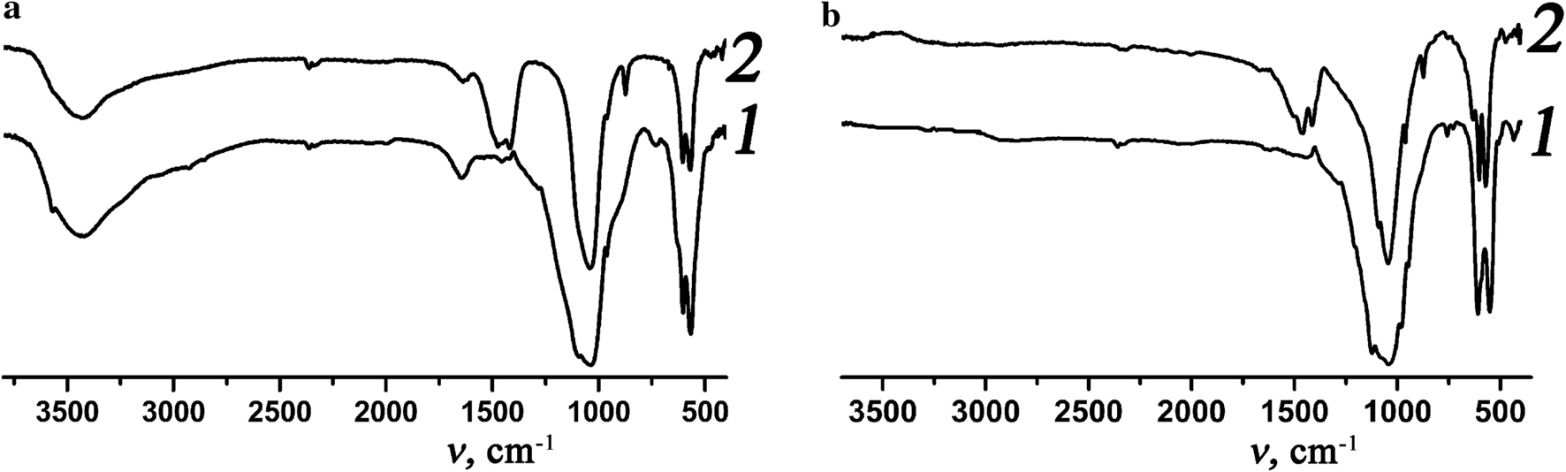
FTIR-spectra for samples III (curves 1) and IV (curves 2) dried at 80 °C (**a**) and heated to 700 °C (**b**)

At the same time, FTIR spectra of all prepared samples are similar and exhibit characteristic bands of phosphate groups in the ranges 560–600 cm^−1^ (ν_4_) and 1000–1100 cm^−1^ (ν_1_ and ν_3_) (Fig. 2a). The broad band in the region 3200–3600 cm^−1^ is attributed to sorbed water and its corresponding deformation vibrations are at 1600 cm^−1^. For samples obtained at molar ratio CO_3_^2−^/PO_4_^3−^ = 1 (samples index II and IV) the bands at 880–870, 1400–1500 cm^−1^ belonging to CO_3_^2−^-groups are also observed (Fig. 2a).

According FTIR spectroscopy data, obtained powders contained different amount of sorbed water that’s why all samples were annealed at 400 °C for 1 h for elimination of sorbed water with aim to determinate their elemental content. As it was shown in reported papers, such heating does not significantly affect particles characteristics [18, 19]. The EDX and CHN data for these samples showed that presence of carbonate in the initial solution caused not only including of carbonate to the composition of precipitates but increasing of amount of alkaline metals (Table 1).

The prepared samples were heated to 700 °C for determination of phase composition. The formation of impurities was not observed. XRD data for these samples showed influence of molar ratio CO_3_^2−^/PO_4_^3−^ in an initial solution on type of crystalline phases (Fig. 1b, e, curves 2). Thus, in the case of prepared samples without carbonate in solution the whitlockite phases were obtained while at the ratio CO_3_^2−^/PO_4_^3−^ = 1 the complex substituted apatite-related calcium phosphates formed. Calculated lattice parameters are depicted in Table 2. Analysis of obtained data showed that values of cell units for prepared sodium-containing whitlockite are between corresponding values for known pure β-Ca_3_(PO_4_)_2_ (*a* = 10.429 Å, *c* = 37.38 Å) [34] and NaCa_10_(PO_4_)_7_ (*a* = 10.4391(1) Å, *c* = 37.310(1) Å) [35] that as and EDX data confirms incorporation of sodium atom in structure of calcium phosphate. At the same time, the calculated parameters for prepared calcium phosphate in potassium-containing solution are almost the same as for β-Ca_3_(PO_4_)_2_ [34] that additionally confirms the absence of K (Table 2). For both apatite-related phases the calculated parameters are some less than corresponding values for Ca_10_(PO_4_)_6_O (*a* = 9.432 Å, *c* = 6.881 Å) that can be caused the partial substitution of calcium atom by alkaline metals and phosphate by carbonate (Table 2). The last fact additionally is confirmed by FTIR spectroscopy (Fig. 2b). The bands at 880–870 and 1400–1500 cm^−1^ which belong to vibration of carbonate groups, confirm B-type substitution of PO_4_^3−^ on CO_3_^2−^ in apatite structure. It should be noted that the broad band in the region 3200–3600 cm^−1^ that corresponds to sorbed water is absent and relative intensity of CO_3_^2−^-group vibrations decrease comparing with corresponding bands for dried at 80 °C samples.

The results of SEM investigation for annealed samples showed the aggregation of particles and increase of their size to 80–300 nm. It should be noted that hexagonal shape of individual particles was kept for sample IV (apatite-related) while sintering to form ceramic particles was observed for sample III (whitlockite-related) (Fig. 1c, f).

Taking into account our early reported data [18, 19] and summarizing above mentioned, synthesized calcium phosphates are characterized by elimination of incorporated water and partial carbonate loss at heating to 700 °C.

Thus, two whitlockite-related (one Na-containing and one pure β-Ca_3_(PO_4_)_2_) and two apatite-related (Na^+^,CO_3_^2−^- or K^+^,CO_3_^2−^-containing calcium phosphates) were used as an initial materials for investigation of immobilization of cesium.

### Characterization of samples after sorption of Cs from aqueous solutions

For purpose the incorporation of cesium ion in the crystal structure of calcium phosphate (whitlockite and apatite-related) the samples after sorption were heated to 700 °C and characterized. According to XRD results, the phase composition of these samples depends on the type of initial calcium phosphate matrix (apatite or whitlockite) and nature of alkaline metals (potassium or sodium) (Table 3).

**Table 3:** Chemical and phases composition of obtained complex substituted calcium phosphates after sorption and heating to 700 °C depending on both type of initial matrix and concentration of cesium ion in the solution

Samples index	Matrix for sorption	C(Cs^+^) in solution, mol L^−1^	Elemental composition (wt%)			Phase composition
			Cs	Ca	P	
1	I	0.05	0.51	36.36	20.69	α-Ca_3_(PO_4_)_2 _+ Apatite
2		0.1	1.55	36.09	21.82	
3		0.25	4.11	41.44	22.91	
4	II	0.05	0.61	38.36	21.39	Apatite
5		0.1	1.31	41.87	22.93	
6		0.25	2.71	41.68	21.14	
7	III	0.05	0.75	41.97	22.04	Whitlockite
8		0.1	2.05	41.75	21.86	
9		0.25	4.94	41.20	21.36	
10	IV	0.05	0.64	44.95	22.87	Apatite
11		0.1	1.43	43.90	21.67	
12		0.25	3.55	45.90	21.17	

For samples 1–3, the presence of cesium changed the general principle of calcium phosphate transformation under annealing to 700 °C. The mixture of α-Ca_3_(PO_4_)_2_ (ICDD # 00-070-0364) and apatite-related phases was obtained while for initial sample the heating to 700 °C caused the formation whitlockite-related calcium phosphate (Table 3). It was found a trend to increase of apatite phase amount to 30% wt for samples obtained with the biggest content of cesium (C(Cs^+^) = 0.25 mol L^−1^) in the initial solution. The calculation of cell parameters for both phases showed that parameters for α-Ca_3_(PO_4_)_2_ (monoclinic system, space group *P2*_*1*_*/a*, *a* = 12.887 Å, *b *= 27.281 Å, *c* = 15.219 Å, β = 126.2°) are close to corresponding literature data (ICDD # 00-070-0364) while the growth of *a* parameter for apatite phase comparing with corresponding for Ca_10_(PO_4_)_6_O (hexagonal system, *a *= 9.432 Å, *c* = 6.881 Å, ICDD # 00-089-6495) was found. The latter indicates the incorporation of Cs^+^ only in composition of apatite-related phase. Thus, presence of Cs^+^ in the powders of Na-containing calcium phosphate caused the stabilization of apatite-type structure.

For samples 7–9 and for initial phosphate (matrix III) the whitlockite-type calcium phosphates were obtained (Table 3). It was found that values of cell parameters for these phases depend on cesium concentration in an initial solution at sorption that indicates the correlation between amount Cs^+^ in the solution and composition of samples after sorption (Tables 3, 4). The increasing of parameter *a* and decreasing of *c* was observed for obtained phosphate at the most concentration of Cs^+^ in solution (sample 9). It should be noted the similar changing of both parameters for cesium-containing phosphate CsCa_10_(PO_4_)_7_ (space group *R3c*, *a *=10.5536(5) Å, *c *= 37.2283(19) Å [36]) comparing with a pure β-Ca_3_(PO_4_)_2_ (*a* = 10.429 Å, *c* = 37.38 Å). This fact indicates that heating of this sample at 700 °C led to incorporation of sorbed cesium in whitlockite-type structure of calcium phosphate.

**Table 4:** The unit cell parameters of obtained complex substituted calcium phosphates after sorption and heating to 700 °C

Samples index	*M*	Lattice parameters		Structure type
		*a*, Å	*c*, Å	
1	Na	9.509(8)	6.829(3)	Apatite
3		9.426(6)	6.843(3)	
7	K	10.422(1)	37.366(2)	Whitlockite
9		10.463(6)	37.349(4)	
4	Na	9.393(1)	6.864(1)	Apatite
6		9.422(1)	6.891(1)	
10	K	9.390(3)	6.878(2)	Apatite
12		9.443(5)	6.892(4)	

For both Na^+^,CO_3_^2−^- and K^+^,CO_3_^2−^-containing calcium phosphates (samples II and IV) the sorption of Cs^+^ and further their heating to 700 °C didn’t change the general scheme of their transformation in crystalline apatite-type phases. Increasing of parameter *a* for obtained phosphates at the most cesium amount in the solution indicates the incorporation of cesium in the apatite-type structure of complex substituted calcium phosphate.

The amount of Cs^+^ in obtained powders was determined using both EDX and X-ray fluorescent methods. It was found the correlation between obtained results which showed that content of cesium in prepared composites and apatites increase with growth of its concentration in the initial solutions (Table 4). It should be noted that the most amount of cesium was found in whitlockite-related phases obtained at the largest concentrations of Cs^+^.

**Fig. 3:**
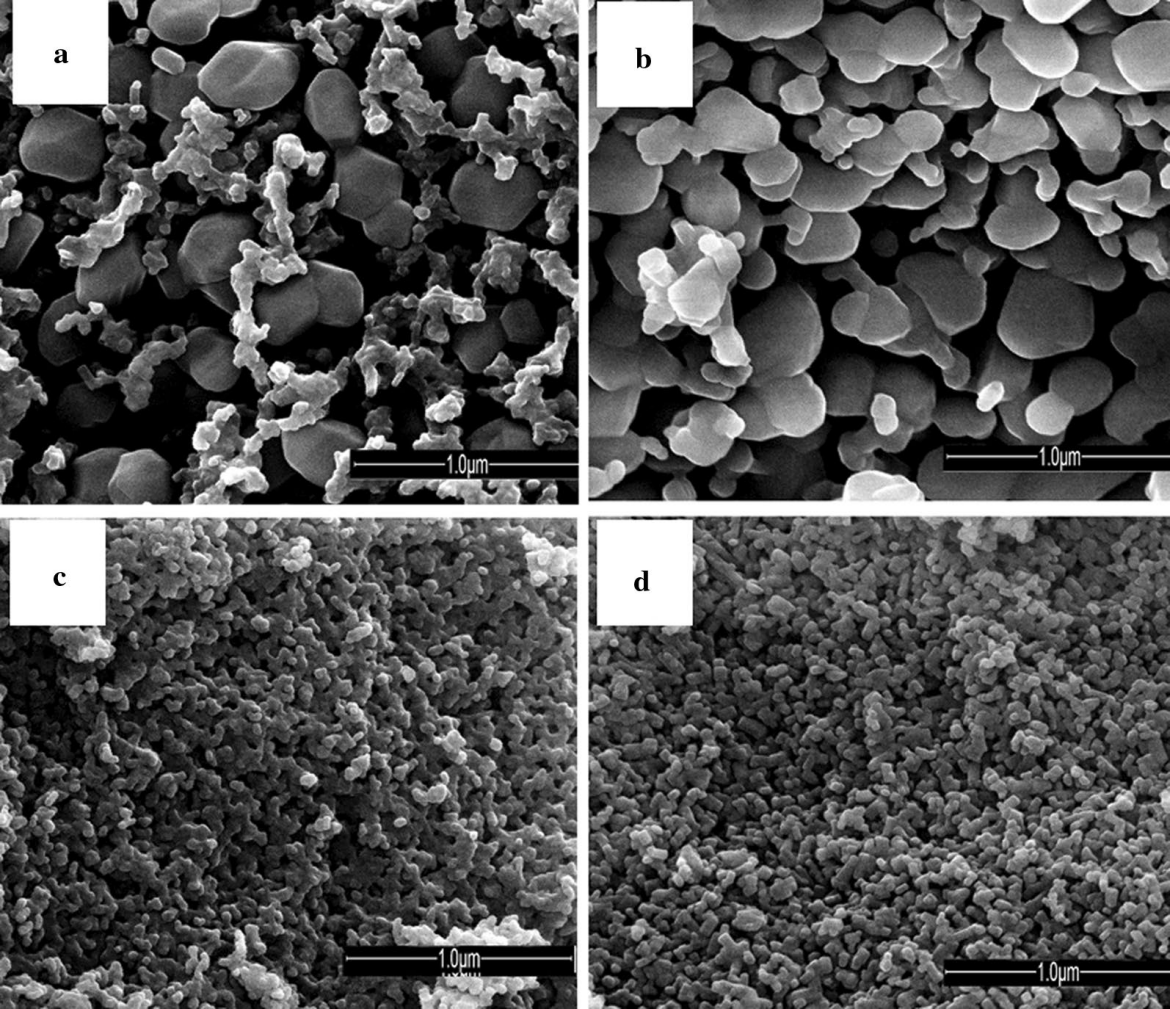
SEM images of samples 3 (**a**), 9 (**b**), 6 (**c**) and 12 (**d**) heated to 700 °C

SEM picture for sample 3 showed two types of particles that correlated with the XRD analysis, namely the formation of mixture of α-Ca_3_(PO_4_)_2_ (ICDD # 00-070-0364) and apatite-related phases (Fig. 3a). The first phase formed in the hexagonal shape particles with size to 400 nm while apatite particles aggregated with formation of dense grains which amount is less.

It was found that the presence of Cs^+^ in other samples did not significantly affect particles morphology with conservation of spherical shape and size of particles (Figs. 1f, 3c, d) or formation of more compact grains (Figs. 1c, 3b) for apatite- or whitlockite-related complex substituted calcium phosphates, respectively.

**Fig. 4:**
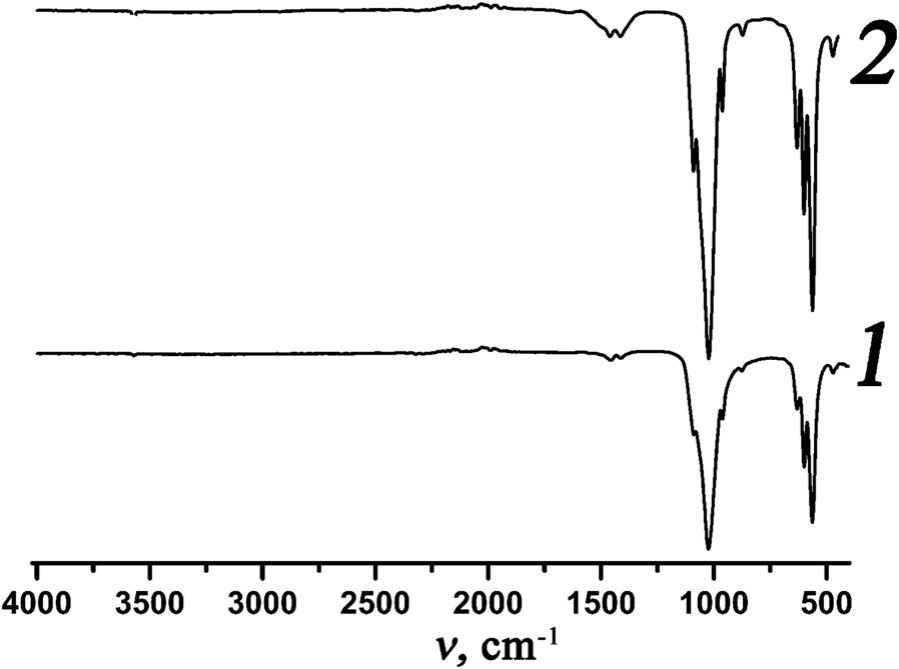
FTIR-spectra for samples 6 (curve 1) and 12 (curve 2) heated to 700 °C

Additionally, it was found that presence of cesium resulted in increasing of stability of carbonate-group in apatite structure for samples 6 and 12 (Table 4). Thus, according to CHN analysis, after heating of matrixes II and IV to 700 °C the content of C was 0.09 and 0.62 wt%, respectively, while for samples 6 and 12 corresponding amounts were 0.36 and 0.74 wt%. This fact indicates about stabilization of carbonate group in apatite structure under influence of bigger alkaline ions such as potassium and cesium. The presence of carbonate groups in obtained M, Cs^+^-containing apatites was confirmed by FTIR-spectroscopy (the bands at 880–870, 1400–1500 cm^−1^) (Fig. 4). The relative intensities of such modes are higher for sample 12 than for sample 6 that correlates with CHN analysis data.

Obtained results indicate the perspective of using of nanoparticles of synthetic complex substituted calcium phosphates for removed of Cs^+^ from aqueous solution. It is known that the ability of phosphate to bind metal ions depends on its structure and chemical composition, specific surface area, and also the nature of metal ion. In the case of apatites, sorbed metal ions can be bound at the surface (adsorption) or exchanged of atoms in the structure (ion exchange). Dissolution of calcium phosphate and the formation of new metal phosphate phases is also possible (dissolution–precipitation method). Among known mechanisms of including of atoms in structure of calcium phosphate the ion exchange and reprecipitation of a partly substituted phosphates are the most desirable because these processes result in the formation of the more stable product [37, 38]. In our determined systems the cesium is sorbed by nanoparticles of calcium phosphate and then at heating to 700 °C filled the cationic vacancies in a nonstoichiometric calcium phosphates. In this way the immobilization of cesium in stable apatite and whitlockite-related materials takes place.

## Conclusions

The particularities of cesium ion incorporation in different types (apatite or whitlockite) structure of calcium phosphates were investigated. The nanoparticles of calcium phosphates as initial material for sorption were obtained by wet precipitation from aqueous solutions and characterized. Obtained results showed the influence of molar ratio of CO_3_^2−^/PO_4_^3−^ in initial solution on composition and structure-type of prepared calcium phosphate. Thus, addition of carbonate in solution caused to precipitate of calcium phosphate that transformed in complex substituted apatite at heating to 700 °C.

Based on combination of X-ray diffraction and chemical analyses for samples after sorption, it was demonstrated that the amount of Cs^+^ in obtained calcium phosphates increased with its concentration in the initial solution. The biggest amount of cesium was found in case of the potassium-containing whitlockite-related phosphates that may indicates about influence of chemical composition and type of initial matrix on sorption possibility of complex substituted calcium phosphates. Except this, for sodium-containing sample it was found that even minor amounts of Cs^+^ in its composition significantly changed the general principle of its transformation under annealing to 700 °C with formation the mixture of α-Ca_3_(PO_4_)_2_ and apatite-related phase that contains Cs^+^.

Obtained results indicate on possibility of using of nanoscale synthetic apatite and whitlockite-related calcium phosphates for development of approaches to remove of cesium from nitrate solution. The further heating of sorbed samples to 700 °C allows to immobilize of Cs^+^ in stable crystallized materials for its storage.
